# A mixed-methods analysis of patient safety incidents involving opioid substitution treatment with methadone or buprenorphine in community-based care in England and Wales

**DOI:** 10.1192/j.eurpsy.2021.1488

**Published:** 2021-08-13

**Authors:** R. Gibson, N. Macleod, L. Donaldson, H. Williams, A. Sheikh, A. Carson-Stevens

**Affiliations:** 1 Community Mental Health Team, Livewell Southwest, Plymouth, United Kingdom; 2 Division Of Population Medicine, Cardiff University, Cardiff, United Kingdom; 3 Department Of Non-communicable Disease Epidemiology, London School of Hygiene and Tropical Medicine, London, United Kingdom; 4 School Of Medicine, University of Edinburgh, Edinburgh, United Kingdom

**Keywords:** Opioid, Patient safety, Mixed methods

## Abstract

**Introduction:**

There is a paucity of knowledge and understanding of medical error in opioid substitution treatment programmes.

**Objectives:**

To characterise patient safety incidents involving opioid-substitution treatment with methadone or buprenorphine in community-based care to identify the sources and nature of harm, describe and interpret themes and use this qualitative analysis to identify priorities to focus future improvement work.

**Methods:**

We undertook a mixed-methods study examining incidents involving opioid substitution treatment with methadone or buprenorphine in community-based care submitted between 2005 and 2015 from the National Reporting and Learning System, a repository of incident reports from England and Wales. We analysed each report using four frameworks to identify incident type, contributory factors, incident outcome and severity of harm. Analysis involved detailed data coding and iterative generation of data summaries using descriptive statistical and thematic analysis.

**Results:**

2,284 reports were identified. We found that most risks of harm came from failure in one of four processes of care delivery: prescribing opiate-substitution (n=151); supervised dispensing errors (n=248); non-supervised dispensing errors (n=318); and monitoring and communication activities (n=1544). Most incidents resulting in harm involved supervised or non-supervised dispensing (n=91/127, 72%). Staff- (e.g. mistakes, not following protocols) and organisation-related (e.g. poor working conditions or poor continuity of care between services) contributory factors were present for over half of incidents.

**Conclusions:**

We have identified four processes of care delivery and associated contributory factors, which represent potential target areas for healthcare systems worldwide to develop interventions to improve the safe delivery of opioid substitution treatment.

